# Sulphurous Acid in the Treatment of Pyrosis Is Strongly Recommended by Dr. Lawson

**Published:** 1868-12

**Authors:** 


SULPHUROUS ACID IN THE TREATMENT OF PYROSIS is strongly recommended by Dr. Lawson. *In every instance, he asserts, in which it has been employed, it has, in a very short time, completely arrested the water-brash secretion.* It checks the excessive secretion, stops the vomiting, and lessens the epigastric dragging pain so often complained of. Dr. L. considers, provisionally, that its good effects are due to the production of ozone and the destruction of vegetable germs.

The doses of the acid (B. P.) vary from *m*xxx to ʒj three times a day, shortly before meals. Bitter infusions may be employed as a vehicle, but plain distilled water is best.—*The Practitioner*, September, 1868.

